# Medicine and the Coming South

**Published:** 1914-08

**Authors:** 


### Medicine and the Coming South.

Fiction often lives too long, and the knowledge of the truth spreads slowly; this is notably true of the South. The ante-bellum South, the war-time South, the South of reconstruction years, the South of today, and the coming South all differ, in medicine as in other things. The fiction-conceived ideas of the South are no longer true; the old is being forgotten; the South is living the life of the vital now and looking hopefully and courageously into the face of the future.

Of no other section of the country is it more true that the hope of medicine is bound up in material advancement and its bounds measured by industrial and agricultural progress. That these promoting factors are ever pressing on is the hope of Southern medicine. The great agricultural awakening, the extension of railroads and turnpikes, the iron and cotton industries, the wonderful hydro-electric development, the opening up of harbors, municipal improvements, better banking facilities and the building up of its own wholesale mercantile establishments have all helped the South and Southern medicine. One who has not recently traversed the South can have little idea of the great material strides that wonderful section is now making. The coming South will be a truly great domain.

But as we write in beautiful Santa Barbara by the Sea, a newer Southland, the thought intrudes that the California physicians' offices we have seen in the last few days are just as up-to-date as are the banks, the hotels and the business offices of the West; they have an every-ready air, a completeness and an obvious utilitarian aspect that creates public confidence and engenders business. This is not quite so true of the South. True, in the modern office buildings of the larger Southern cities some fine suites of medical offices are seen; but the medical offices in most Southern places have an example before them in the West, where it has been abundantly demonstrated that good housing and well-appointed facilities are profitable to the doctor.

It is no longer wise to trust so much in the personal element in medicine. "The man behind the gun" still counts, but he must have a modern gun; the old weapons and equipment no longer serve. Really, this Western Coast is setting a pace for more of the country than the South alone. We cannot but compare two instrument houses we have recently visited, one on the Atlantic seaboard and the other on the Pacific. From a superficial view of the two stocks we are led to believe that fully a third of the Eastern stock would be considered as junk in the Western establishment; but the point is, a large Southern stock we looked over was loaded up with these Eastern "stickers."

From the strictly professional standpoint the South always did rank high. One is impressed with dignified and earnest schools like Tulane University and its record of real achievement. Some of the Southern hospitals are models in their way. Atlanta promises to be, in time, a great medical center. The University of Virginia, as well as several other Southern institutions, have high ideals and are doing good and earnest work upon a basis of *professional attainment, not* of large money endowments.

We are impressed with a feeling that medical politics does not figure so largely in the South as elsewhere, and the Southern Medical Association, an earnest and rapidly growing organization, is to be largely credited with this state of affairs. Comparisons are usually odious; but, in entire friendliness, we wish to say that the American Medical Association has somewhat to learn from the Southern Medical Association. This latter Association is balanced, temperate and just in its policies; and its almost phenomenal growth of late testifies to the fact that it is filling a place in the South the A. M. A. has partially failed to fill. The *Southern Medical Journal,* the organ of the Southern Medical Association, is a credit not only to the South but to American medical journalism. Now if the Southern Medical Association can continue as it is, avoiding the pitfalls of politics and sectionalism, the South is destined to *really count* in the determination of national medical policies.

On the other hand, the South has some weak medical schools that could honorably disband or merge without any detriment to the interests of Southern medicine. Lack of financial support weakens a number of necessary Southern hospitals. It is to be hoped that wealthy men will remember these worthy institutions. The South has been charged with overdoing sectarian organization in its educational and hospital foundations. Superficially this appears to be true, but what else could the South do; to whom else could she turn?

Sanitation is a great Southern problem. Go through the many cemeteries of New Orleans, look up its history of yellow fever epidemics; then inspect its wonderful new drainage, sewage, and water systems, as we have done, and you will have an object lesson in what modern sanitation can do. There is much yet to be done; but *dollar* for *dollar* we challenge any other section to show such great sanitary accomplishment as is that of the South.

Southern business men are turning away from sectionalism, and the Southern medical profession is advancing quite as much along broad, nation-blending lines as is any other section, and more than some. The South is distinctly American and is destined to remain so.

The great advantage possessed by the South over the West is that it has avoided frenzied finance and has not boosted land values and taxes. There is too much inflation in parts of the West, notably in California, where high-priced land, large transportation charges, irrigation expenses, a scarce labor market and high taxes have rendered the promotion of agricultural projects one threatening a very narrow margin of profit. This makes many California rural sections poor places in which to get ahead in medicine unless the doctor engages in some side business, as many physicians do in those regions. In the South one is impressed with the fact that the average physician is devoting his whole attention to medicine.

One reads a great deal about the splendid land of the West, and much of it really is very fertile indeed; but the South has good land also. The Shenandoah Valley of Virginia is probably the finest apple section in the United States, the Norfolk trucking section is unexcelled, the Carolinas and Georgia are developing splendid fruit sections and Southern Louisiana has as fine land as we have ever seen anywhere. The grain and cattle industries of the South promise to become immense, in time. And the beauty of it all is that land values are upon an equitable basis, one almost *assuring* a good profit to agricultural enterprises.

These things are well to remember in selecting a location or planning a new home. The doctor of slender resources will have "hard sledding" in establishing himself in the far West; but the South presents many openings where a home can be cheaply purchased, and living expenses will be low while building up a practice.

Florida is the Southern State advertised the most, and that has resulted in an influx of physicians; there are too many doctors *now* in many Florida towns, and they are having rather a hard time of it. As to a physician going to Florida to raise oranges and practice medicine as well, our advice is to do *one* of these things, if you go there, but don't try both at once. Growing oranges is in elaborate horticultural specialty and one not readily mastered. It is easy to dream. The place "where everlasting spring abounds and never withering flowers" does not exist on this earth—except in exploitation literature. It is well for doctors to remember this. All over the North and East one finds discontented doctors dreaming over exploitation literature and longing to move to Florida or California. While Florida and California really do possess great advantages, we can't find in either of those States many Northern and Eastern doctors *realizing upon* their exploitation literature dreams. For the doctor of ordinary financial resources, who is intent upon combining a limited practice with some form of agricultural enterprise, we commend the South, especially as negro labor is cheap and plentiful. After all is said and done, plowing and seeing patients rarely mix well. In the South the doctor can *hire* the work done in his fields and not go bankrupt.

Social conditions in the South are good; better than many Northern people imagine. The truth is "the Doctor" is a social factor, and is respected as a leader in the community down South.

It is perfectly true, though, that in the Southern seaports, vice is very much in evidence, obtrusively so in New Orleans. Such things, however, are very definitely segregated; and is there any seaport town the world over where such conditions do not exist?

But they are harder to find in New York and Philadelphia and the good people do not merely shrug their shoulders and leave it all to law-forgetting policemen.

Southern physicians need to wake up and clean up these "red light districts" in their seaport cities or the coming generation will present some problems.

The smaller Southern communities seem to be more correct in morals than is common in other portions of the nation, and they have it in their own hands to cleanse the plague spots in their seaports.

Medical selfishness and medical rings do not flourish in the South as they do in some places, notably in the West. In truth, some Western States have medical examining boards whose chief function is not at all the protection of the public against the quack, but rather the protection of their own little "crowd" against the honorable medical profession at large. This is a form of peanut politics of which the South is not guilty, be it said to its credit. The result is the West has vastly more quacks than has the South.

We took occasion to interview physicians connected with the Federal service in Washington, and there is an intense feeling of disgust there over the narrow State "police power" idea of the medical profession and a conviction that something along the line of Federal regulation of medical practice is due. The South appears to share in this feeling. The South deprecates present-day commercializing of medicine, and it takes a high-plane view of medical ethics, a form of ethics binding upon the "big men" as well as the rank and file.

Organized medicine is not as strong in the South as it ought to be, but the South can hardly be blamed for this. Whether rightly or wrongly, an immense number of Southern physicians feel that National and State medical societies are not properly organized. But Southern sentiment in the matter is not sufficiently concrete to propose a remedy. Just what should be done to promote organized medicine in the South we do not know; but something should be done. Many Southern physicians are financially poor; they lack the advantages of co-operation. It seems to us the Southern men should get together and determine what needs to be done to advance the interests of the Southern profession. Fees are too low in much of the South, and many physicians are working very hard for very little money. They seem to be an unselfish lot down there and not much inclined to make a fight for better conditions.

With the wonderful future awaiting the South and the earnest character of its physicians, we can't help but feel that greater rewards are due to the Southern physician than he is now receiving. He has too big a proportion of charity work to do; he is not enough in the medical lime-light; he labors under too many discouragements for so good a district of the country, and he is not saving much money. Our own thought is that the Southern Medical Association can do much for him. If it becomes the power it promises to become, perhaps it can take up intelligently with our National and State medical societies the questions confronting the medical South. And, on the other hand, the high and unselfish aims and tone of medicine in the South is needed to balance the councils of our National medical conferences. In due time medicine will be federalized in the United States. When that time comes, the South will probably have much to say.—*The Medical Council.*

